# *Haloferax volcanii* as immobilised whole cell biocatalyst: new applications for halophilic systems

**DOI:** 10.1007/s00253-019-09725-y

**Published:** 2019-03-15

**Authors:** R. U. Haque, F. Paradisi, T. Allers

**Affiliations:** 1 School of Life Sciences, Queens Medical Centre, University of Nottingham, Nottingham, NG7 2UH UK; 2 School of Chemistry, University Park, University of Nottingham, Nottingham, NG7 2RD UK

**Keywords:** *Haloferax volcanii*, Biocatalysis, Biotransformation, Whole cell immobilisation, Biocatalyst

## Abstract

Enzyme-mediated synthesis of pharmaceutical compounds is a ‘green’ alternative to traditional synthetic chemistry, and microbial engineering opens up the possibility of using whole cells as mini-factories. Whole-cell biocatalysis reduces cost by eliminating expensive enzyme purification and cofactor addition steps, as well as resulting in increased enzyme stability. *Haloferax volcanii* is a model halophilic archaeon encoding highly salt and organic solvent tolerant enzymes such as alcohol dehydrogenase (*Hv*ADH2), which catalyses the reduction of aldehydes and ketone in the presence of NADPH/NADH cofactor. A *H. volcanii* strain for constitutive *Hv*ADH2 expression was generated using a strong synthetic promoter (p.*syn*). The strain was immobilised in calcium alginate beads and repeatedly used as a whole-cell biocatalyst. The reduction of acetophenone, used as test substrate, was very successful and high yields were detected from immobilised whole cells over repeated biotransformation cycles. The immobilised *H. volcanii* retained stability and high product yields after 1 month of storage at room temperature. This newly developed system offers halophilic enzyme expression in its native environment, high product yield, stability and reusability without the addition of any expensive NADPH/NADH cofactor. This is the first report of whole cell–mediated biocatalysis by the halophilic archaeon *H. volcanii*.

## Introduction

**Fig. 1:**
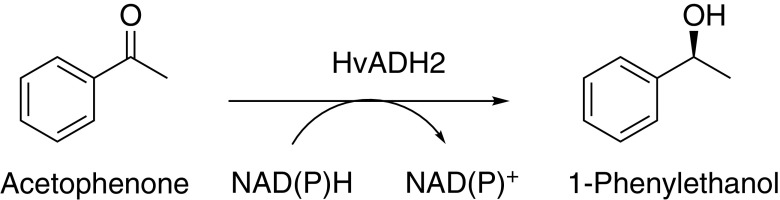
Bioconversion of acetophenone to 1-phenylethanol catalysed by *Hv*ADH2. In the presence of NADPH/NADH cofactor, acetophenone is reduced to 1-phenylethol by the activity of *Hv*ADH2 enzyme

Enzymes serve as excellent catalysts due to their ability to catalyse reactions with high enantioselectivity and regioselectivity under environmentally benign conditions (Schmid et al. 2001; Sheldon and Woodley 2018). Due to rapid development of microbial strain engineering and directed evolution, biocatalysis has gained importance in the production of pharmaceutical and agrochemical compounds (Arnold 2018; Bornscheuer et al. 2012; de Carvalho 2017; Schoemaker et al. 2003; Yadav et al. 2012). From an economical and environmental point of view, biocatalysis eliminates the need for blocking and deblocking steps involved in enantio- and regioselective organic synthesis, and the use of fossil fuels needed for achieving high temperature and pressure conditions. Enzymes from extremophiles have the advantage that they are functional in the presence of organic solvents, high temperatures and high salt concentrations (Demirjian et al. 2001). *Haloferax volcanii* is an extremely halophilic archaeon with its origin in the Dead Sea (Mullakhanbhai and Larsen 1975). It is the organism of choice for haloarchaeal genetics due to the availability of extensive genetic tools (Allers 2010; Allers and Mevarech 2005; Allers et al. 2004). It is easily culturable and has simple laboratory growth condition (aerobic and 45 °C). It encodes highly salt and organic solvent-tolerant enzymes such as alcohol dehydrogenase (*Hv*ADH2) (Alsafadi and Paradisi 2013). Purified *Hv*ADH2 has an unusually broad substrate scope and it catalyses the production of industrially valuable chiral alcohols in the presence of NADPH/NADH cofactor (Fig. 1) (Alsafadi et al. 2017; Timpson et al. 2013).

Industrial application of whole cells for biocatalysis has multiple advantages over purified enzymes. Firstly, whole cell biocatalysts are readily and inexpensively prepared, eliminating the need to purify enzymes (Ishige et al. 2005). Secondly, addition of stoichiometric amounts of expensive cofactor such as NADPH/NADH is not necessary since whole cells can regenerate cofactors *in situ* (Devaux-Basseguy et al. 1997; Wachtmeister and Rother 2016). Thirdly, enzyme stability is increased due to protection offered by microbial whole cell compartments, and there is ample scope for repeated uses in subsequent processes (de Carvalho 2017; Ishige et al. 2005; Lin and Tao 2017). For these reasons, whole cells are considered as the cheapest form of catalyst for bioconversion (Tufvesson et al. 2011). Immobilisation of microbial whole cells has been used extensively for production of useful chemicals via biotransformation (Chibata 1979; Gotovtsev et al. 2015; Gungormusler-Yilmaz et al. 2016; Zhu 2007) since it eases microbial handling, provides high cell density and most importantly improves operational stability and reusability (Bickerstaff 1997).

Halophilic proteins typically have many acidic residues on the protein surface to make them soluble in high salt environments (Danson and Hough 1997; Mevarech et al. 2000). This can pose problems for heterologous expression of halophilic enzymes in biotechnologically attractive *E. coli* host cells (Lin and Tao 2017; Zhao et al. 2014), since halophilic proteins aggregate and misfold in low ionic environment. Where halophilic proteins have been expressed in *E. coli,* solubilisation and refolding of insoluble proteins from inclusion bodies has been carried out in a hypersaline environment (Cendrin et al. 1993; Connaris et al. 1999). However, this approach is not universally effective as many enzymes remain inactive (Timpson et al. 2012). Therefore, it is preferable to use a halophilic system to ensure high enzyme expression in their native environment. Such halophilic systems minimise contamination, as other microbes are not able to tolerate the molar salt concentrations. By harnessing the power of microbial engineering and whole cell biocatalysis, we have engineered a halophilic system using immobilised *H. volcanii* whole cells capable of efficient biotransformation through the over-expressed *Hv*ADH2 enzyme activity without the addition of expensive NADPH/NADH cofactor.

## Materials and methods

### Reagents, strains and growth conditions

All chemicals and reagents were purchased from Sigma-Aldrich (UK) unless otherwise stated. Restriction enzymes and DNA ligases were bought from New England Biolabs (USA). *H. volcanii* strains were grown at 45 °C on casamino acid (*Hv*–Ca) agar or complete (*Hv*-YPC) agar, and in *Hv*-YPC broth, and growth media and 18% salt water were prepared as described previously (Allers et al. 2004). 2 × YPC^+^ broth was prepared using 10 g/L yeast extract, 2 g/L casamino acid, 2 mM K_2_HPO_4_/KH_2_PO_4_ at pH 7.5, 5 mM NH_4_Cl and 0.5% (*v*/*v*) lactate (Strillinger et al. 2016). All *H. volcanii* strains are derivatives of H1325 (Timpson et al. 2013), the genotype of H1325 is *ΔpyrE2 ΔhdrB Nph-pitA Δmrr Δadh2 Δadh1*.

### Construction of expression plasmid pTA1992 containing p.*syn* promoter

*Bst*BI and *Nde*I digested pTA1392 was gel purified using gel purification kit (MACHEREY-NAGEL, Germany). Oligonucleotides O247 (Sense, p.synF- CGAGAATCGAAACGCTTATAAGTGCCCCCCGGCTAGAGAGAT) and O508 (Antisense, p.synR2-TAATCTCTCTAGCCGGGGGGCACTTATAAGCGTTTCGATTCT) were hybridised to generate the p.*syn* promoter DNA with *Cla*I and *Nde*I-compatible ends. Oligo hybridisation was performed in an Eppendorf tube containing 20 μl of each oligo (10 μM), 10 μl of NEB Buffer 2 and 50 μl dH_2_O at boiling temperature and cooling afterwards. *Bst*BI and *Nde*I digested pTA1392 and hybridised oligos were ligated and transformed into *E.coli* XL-1 Blue by electroporation. Following plasmid extraction (Maxiprep, MACHEREY-NAGEL, Germany) integration of p.*syn* promoter was verified using two sequencing primers (O363: TTAAGTTGGGTAACGCCAGGG) and (O919: AATTCGATATCTCACTTCTCGAACTGCGGGTGCGACCAGCTAGCTGGGGCGCCA). The resulting plasmid was designated pTA1992.

### Construction of expression plasmid pTA2035-*adh2*

A total of 1050 bp long *adh2* (HVO_B0071) was PCR amplified from the pTA1205 plasmid (Timpson et al. 2013) using primers O757 (*adh2*F CACAGCGT**TCATGA**AATCAGCAGTC, *Bsp*HI cut site) and O758 (*adh2*R, GTCT**GGATCC**GGGTGTGTCTTACTCG, *Bam*HI cut site). PCR amplification was performed using the NEB Q5® Hot Start High Fidelity DNA Polymerase. *adh2* was cloned into *Pci*I and *Bam*HI digested pTA1992 plasmid using *Bsp*HI and *Pci*I compatible ends. PCR reactions were purified using PCR purification kit (MACHEREY-NAGEL, Germany) and were digested with *Bam*HI-HF and *Bsp*HI. Plasmid vector pTA1992 was digested with *Bam*HI-HF and *Pci*I. Ligation with T4 Ligase was followed by transformation as above. Primers O245 (HEXTF – GCGCGTAATACGACTCACTATAGGG) and O47 (PBSR2 – CGCGCAATTAACCCTCACTAAAG) were used for sequencing confirmation and resulting plasmid was designated pTA2035.

### Construction of *H. volcanii* strains

All plasmid transformations into *H. volcanii* were performed as described (Allers et al. 2004). pTA2035 was transformed into the H1325 (Timpson et al. 2013) to generate strain H3924 (*ΔpyrE2 ΔhdrB Nph-pitA Δmrr Δadh2 Δadh1*) {p.*syn*::his-tag-*adh2* + pyrE2 + hdrB+}. Empty vector control strain was generated by transforming pTA1992 into H1325 to generate H3925 (*ΔpyrE2 ΔhdrB Nph-pitA Δmrr Δadh2 Δadh1*) {p.*syn*::his-tag pyrE2 + hdrB+}.

### Purification and enzyme activity determination of *Hv*ADH2

H3924 broth culture (330 ml) was grown up to an OD_650nm_ of 1.0. The cell pellet was resuspended in 7 ml of Buffer A (20 mM HEPES at pH 7.5, 2 M NaCl, in dH_2_O) with 1 × EDTA-free SigmaFast protease inhibitor cocktail and was lysed by sonication. Lysate was sequentially filtered through 0.8 μm, 0.45 μm and 0.2 μm filters. A 0.5 ml of 0.2 M NiSO_4_-equilibriated IMAC Sepharose 6 Fast Flow beads were added to the cleared lysate and incubated shaking at 4 °C for 1 h. After loading a Bio-Rad Poly-Prep gravity column, the beads were washed twice using Buffer A + 1 mM PMSF at 4 °C, and protein was eluted in 2 ml of Buffer A + 100 mM Imidazole followed by 2 ml Buffer A + 200 mM Imidazole. Both elutions were combined and concentrated in 2 M KCl, 50 mM Glycine/KOH at pH 8 in a Vivaspin® 20 column (Sartorius, Germany). Protein concentration was determined using a Pierce™ BCA Protein Assay Kit (Thermo Scientific, UK).

*Hv*ADH2 activity was determined by measuring NADPH produced at 340 nm in intervals of 1 min for 20 min at 45 °C using an EPOCH 2 microplate reader (BioTek). Assay was performed in a total volume of 200 μl in 96 Well Clear Flat Bottom UV Transparent Microplate (Corning®, 3635). Reaction mix contained 10 μl of purified *Hv*ADH2 enzyme, 20 μl of ethanol, 2 μl of 1 mM NADP^+^, 168 μl of 4 M KCl and 50 mM Glycine/KOH at pH 10.

### Immobilisation of *H. volcanii* within alginate beads

An *H. volcanii* 380 ml culture (3.5 g pellet) grown up to an OD_650nm_ of 1.0–1.2 and pelleted. In a sterile 50 ml Duran bottle, the cell pellet was gently resuspended in 12 ml YPC broth and mixed with 50 ml of 4% sodium alginate (SIGMA 71238) solution in dH_2_O using a sterile magnetic stirrer. After 20 min, using a 20 ml BD Plastipak^TM^ syringe in a clamp stand, the *H. volcanii*–Na-alginate mixture was added dropwise to 100 ml of 1.5% CaCl_2_ (in H_2_O) solution. The distance between the syringe tip and surface of CaCl_2_ solution was 13 cm. Beads were left to settle for 30 min at room temperature. *H. volcanii* beads were separated from the bulk CaCl_2_ solution using a sterile strainer and washed twice with 18% salt water before biotransformation.

### Biotransformation protocol

For each immobilised *H. volcanii* strain, 20 g of weighed beads were used for biotransformation. After two washes with 18% salt water, beads were added to a sterile 250 ml Duran conical flask with 5 mM acetophenone substrate in 50 ml YPC broth + 4% glucose unless otherwise stated. Flasks were shaken at 150 rpm agitation for 24 h in a 45 °C water bath. After 24 h, 2 ml of supernatant was centrifuged at 18000 RCF for 10 min. Product was measured by HPLC by adding 50 μl of supernatant into a HPLC vial with 475 μl of HPLC grade acetonitrile and 475 μl 0.2% HCl in dH_2_O. Diameter of the beads before starting and after biotransformation for 12 successive cycles was measured using Vernier callipers**.**

### HPLC protocol

HPLC was performed using a reverse phase XBridge C18, 3.5 μm, 2.1 mm × 30 mm column with a flow rate of 0.8 mL/min at 40 °C temperature. Samples were analysed using a gradient of two solutions A (0.01% ammonia acid in water) and B (acetonitrile). Gradient program was used as follows; at 0 min 95% A, 5% B, at 3 min 5% A and 95% B, at 3.10 min 0% A, 100% B, at 3.51 min 95% A, 5% B and at 4.5 min 95% A, 5% B. Retention time for the product (1-phenylethanol) and substrate (acetophenone) was determined as 1.7 min and 2.2 min respectively. UV absorption was measured at 210 nm. Amount of product was quantified using a standard curve for 1-phenylethanol sample gradient.

## Results

### Construction of a constitutive gene expression system in *H. volcanii*

**Fig. 2:**
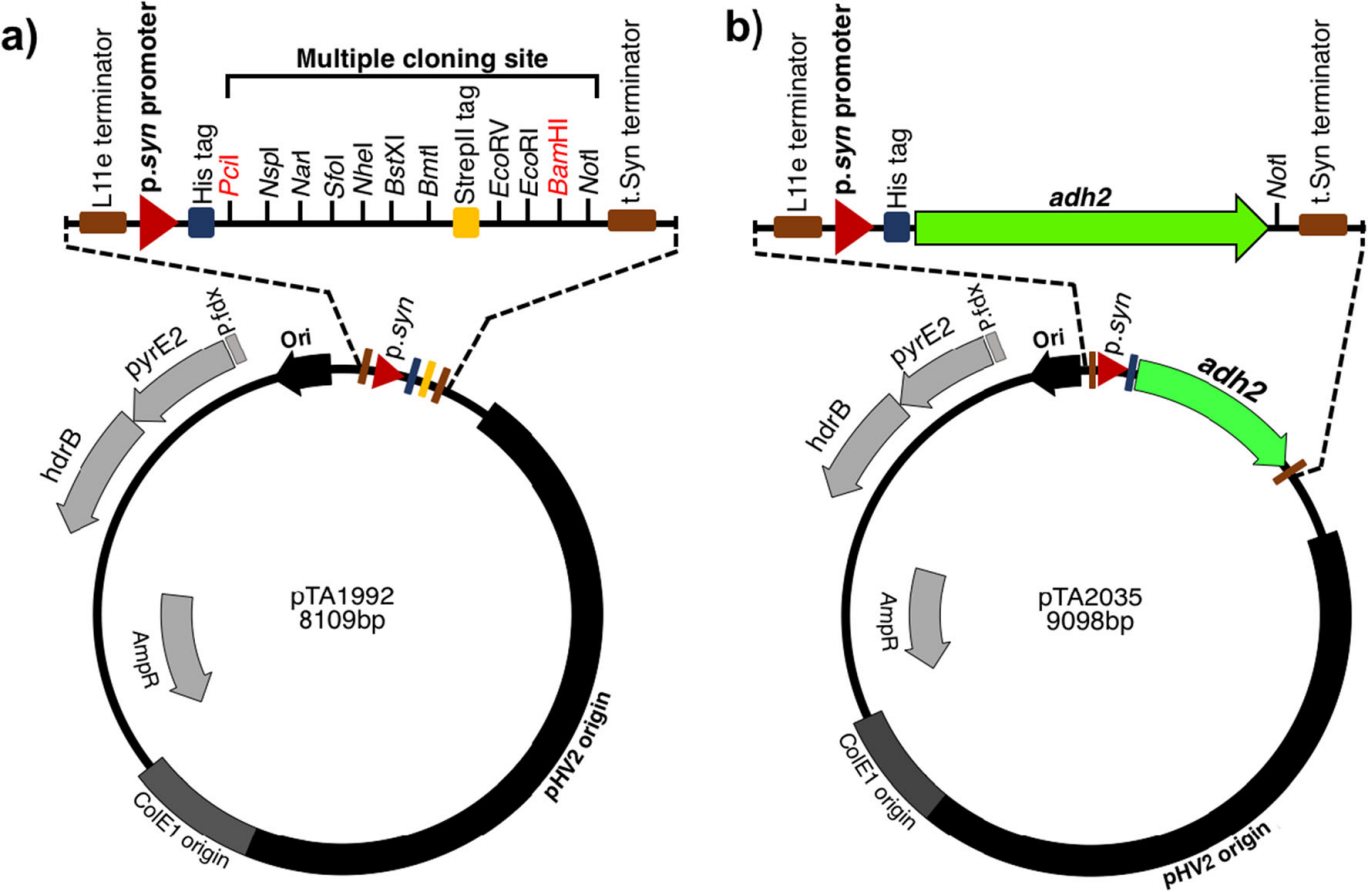
Map of constitutive gene expression system in *H. volcanii.***a** pTA1992. The p.*syn* promoter is used for constitutive expression of His (Histidine) tagged proteins in *H. volcanii.* A multiple cloning site is located after His tag, and is flanked by L11e and t.Syn terminators to prevent read-through transcription. pTA1992 was transformed into H1325 to generate H3925 to serve as empty vector control for biotransformation, **b** pTA2035**.** The *adh2* (HVO_B0071, 1050 bp) gene was inserted in pTA1992 under the control of the constitutive p.*syn* promoter. pTA2035 was transformed into H1325 to generate H3924 for *Hv*ADH2 expression

Existing gene expression systems for *H. volcanii* are based on a tryptophan inducible promoter (p.*tna*) (Allers 2010). For example, the *Hv*ADH2 overexpression strain H1332 requires induction with mM concentrations of tryptophan to activate *adh2* expression (Timpson et al. 2013). To alleviate the need for tryptophan supplementation in batch cultures, a *H. volcanii* strain was generated that expresses *adh2* constitutively. p.*syn* is 43 bp long strong synthetic promoter based on the *H. volcanii* consensus tRNA promoter sequence (Large et al. 2007). A plasmid vector pTA1992 for constitutive gene expression was created by replacing the tryptophan inducible promoter (p.*tna*) of pTA1392 with the constitutive p.*syn* promoter (Fig. 2a). The *Hv*ADH2 overexpression plasmid pTA2035 was generated by cloning the *adh2* under the control of the p.*syn* (Fig. 2b), and the *H. volcanii* strain H3924 was generated by transforming the pTA2035 plasmid into the H1325 (*Δadh2 Δadh1*). It was essential to use *adh*-deleted strain as previous work had shown endogenous ADH form a complex with recombinant ADH (Timpson et al. 2013). The empty vector pTA1992 was transformed into H1325 to generate the background strain H3925 as a control for biotransformation.

### *Hv*ADH2 protein expression and enzymatic activity in H3924

**Fig. 3:**
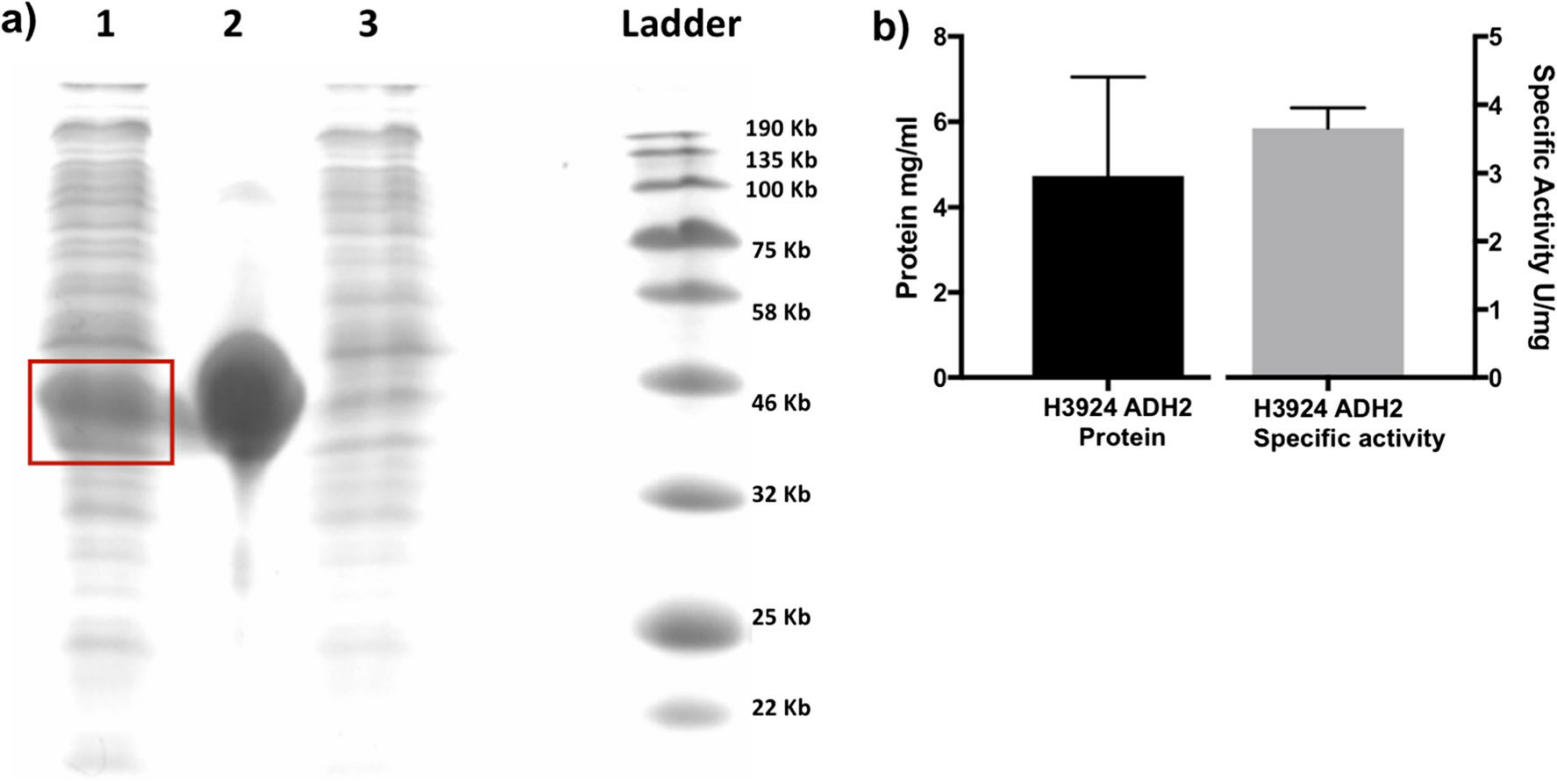
*Hv*ADH2 expression and enzymatic activity from H3924. **a** SDS-PAGE confirmation of high *Hv*ADH2 expression from H3924, lane 1, H3924 His-*Hv*ADH2 cell lysate (*Hv*ADH2 indicated in red box); lane 2, purified *Hv*ADH2 (37.8 kDa); lane 3, H3925 cell lysate, **b** purified *Hv*ADH2 protein concentration and enzyme specific activity, mean ± SD, *n* = 5

To confirm *Hv*ADH2 expression and enzymatic activity in H3924, histidine-tagged *Hv*ADH2 was purified using nickel-based affinity chromatography. SDS-PAGE gel electrophoresis revealed a band corresponding to the subunit molecular weight of 37.8 kDa as expected (lane 2, Fig. 3a) and high levels of *Hv*ADH2 protein production (mean 4.74 mg/ml) and specific activity (mean 3.7 U/mg of protein) were detected in H3924 (Fig. 3b).

### Immobilisation of *H. volcanii* within calcium alginate beads

**Fig. 4:**
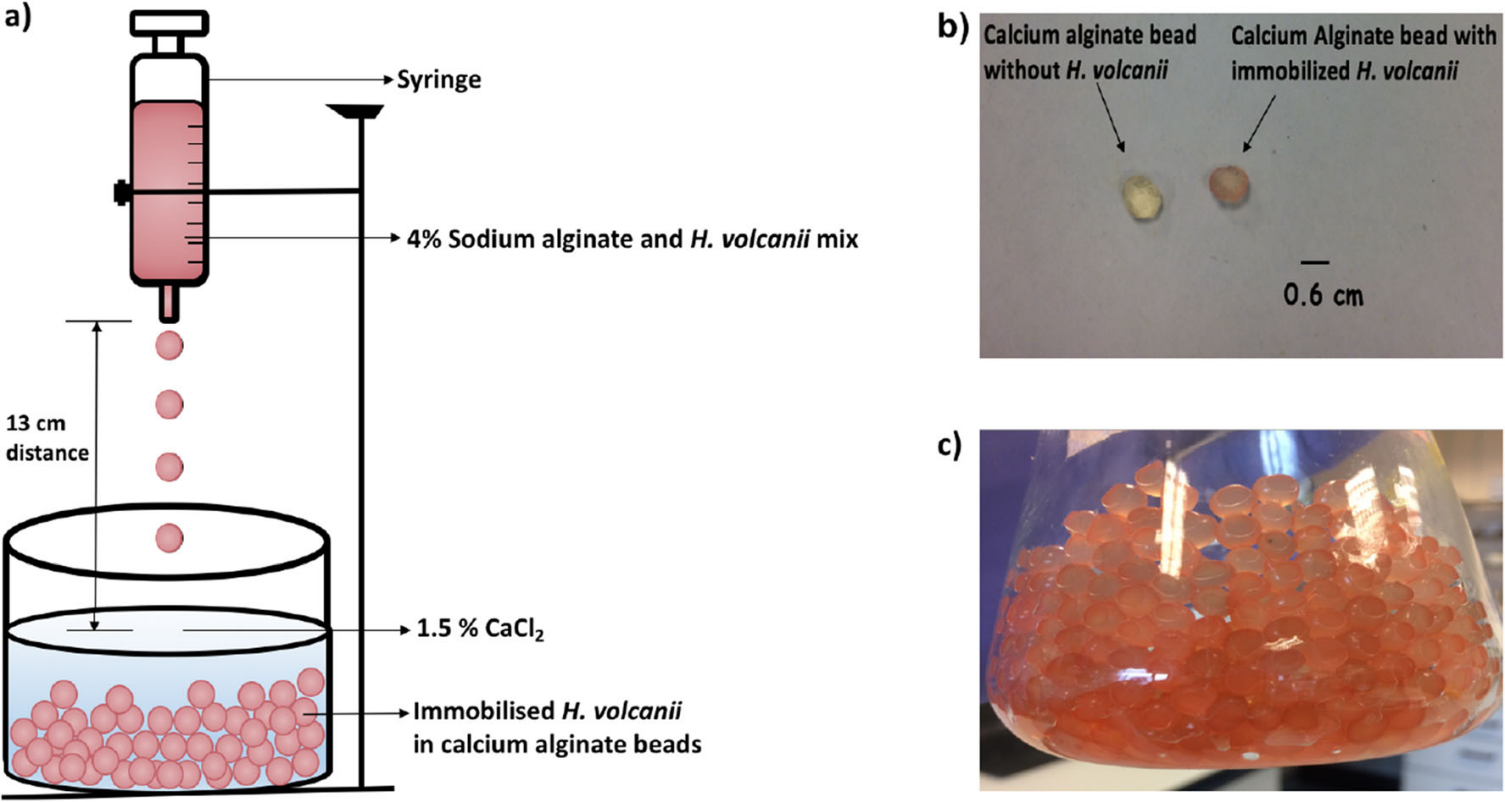
Procedure for immobilising *H. volcanii* within calcium alginate beads. **a***H. volcanii* cell pellet resuspended in YPC broth was mixed gently with 4% sodium alginate solution (in dH_2_O) using a magnetic stirrer. Using a BD Plastipak^TM^ syringe, the mixture was added dropwise into 1.5% CaCl_2_ solution to form beads, **b** and **c** formation of pink beads confirmed entrapment of *H. volcanii* within calcium alginate beads, the distinctive colour is due to presence of high carotenoid pigment

The naturally occurring marine polymer alginate is the most widely used encapsulation agent for cell immobilisation (Strand et al. 2004). Alginate is a linear binary polysaccharide composed of 1-4 linked β-d-mannuronic acid and α-l-guluronic acid residues (Haug and Larsen 1966; Lee and Mooney 2012). When a mixture of cell suspension and alginate is dropped into a solution of divalent cations (Ca^2+,^ Ba^2+^, Sr^2+^), a porous spherical gel matrix is formed instantly through ionic cross-linking between cation and anionic guluronic acid residues of alginate (Melvik and Dornish 2004). The resulting matrix in the form of a bead possesses biotechnologically attractive properties such as instant gel formation, non-toxicity, high porosity and an inert aqueous and heat-stable matrix (Gombotz and Wee 1998; Melvik and Dornish 2004). Calcium alginate beads have been successfully used in biocatalysis to produce 1-phenylethanol from *Hansenula capsulata* (Hasegawa et al. 1998), *Rhodotorula glutinis* (Kurbanoglu et al. 2010) and *Pichia capsulata* (Illeová et al. 2015). The *Hv*ADH2 expressing H3924 strain was immobilised within the calcium alginate beads for 1-phenylethanol production (Fig. 4a) and entrapment of *H. volcanii* was confirmed by distinctive pink carotenoid pigment produced by this organism (Fig. 4b and c).

### Effect of substrate concentration, culture agitation speed, temperature, nutrient media and gene expression system on product yield

**Fig. 5:**
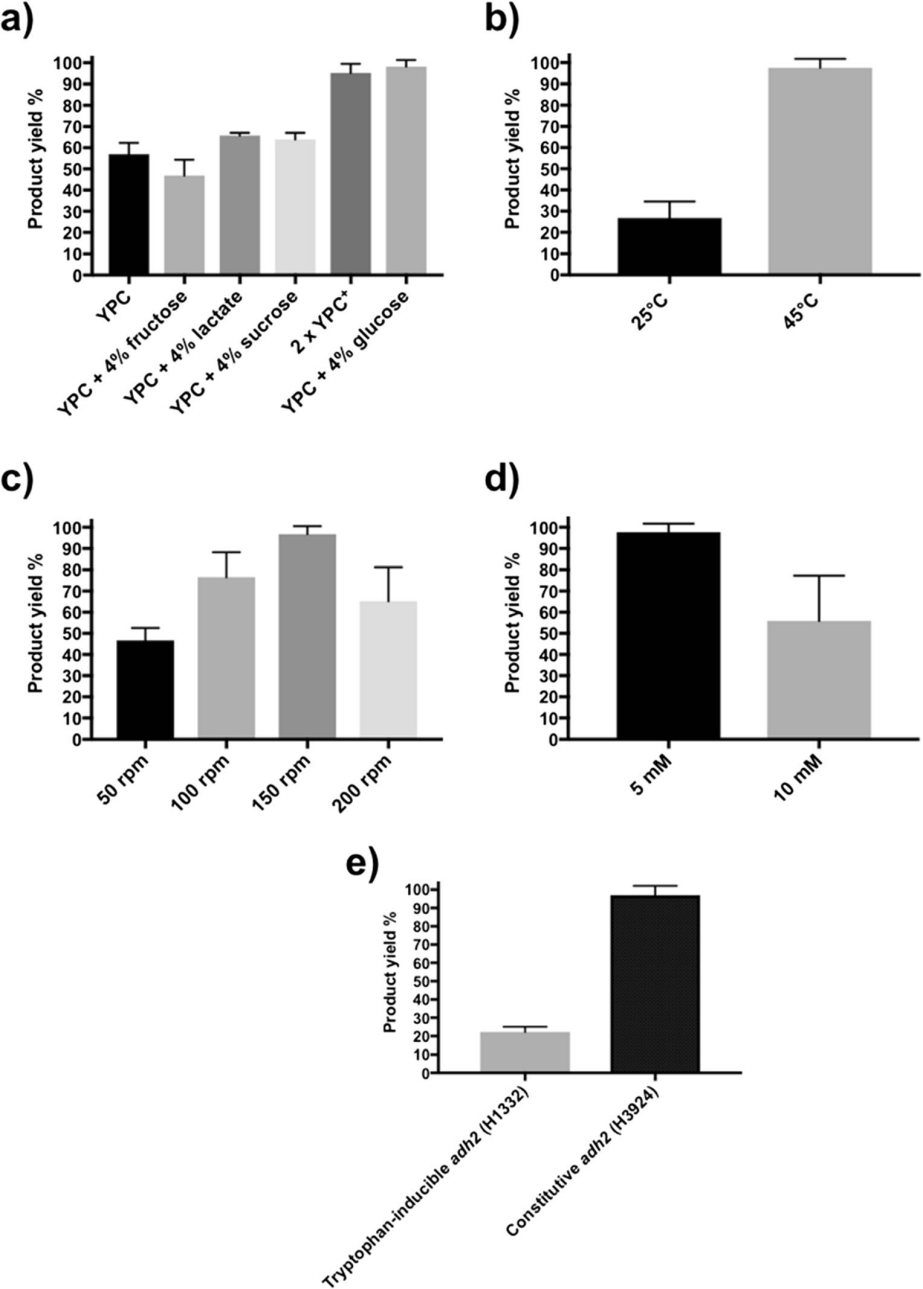
Characterisation of conditions for maximal product yield. **a** Nutrient broth. Biotransformation reactions were performed in YPC, YPC + 4% fructose, YPC + 4% lactate, YPC + 4% sucrose, 2 × YPC^+^, YPC + 4% glucose, *n* = 3. **b** Temperature**.** Biotransformation was performed at 25 °C and 45 °C, *n* = 3. **c** Agitation speed. Beads in broth were agitated at 50 rpm, 100 rpm, 150 rpm and 200 rpm speed, *n* = 5. **d** Substrate concentration. 10 mM and 50 mM acetophenone substrate was added to the broth, *n* = 3. **e** Inducible vs constitutive gene expression system. Biotransformation was performed using the inducible (+ 5 mM tryptophan) *adh2* strain H1332 and the constitutive *adh2* strain H3924, *n* = 3, mean ± SD

The optimal conditions for maximal product yield were determined. Alongside the regular YPC broth used for culturing *H. volcanii*, the impact of different nutrient sources such as fructose, lactate, sucrose and glucose was investigated. In addition, the 2 × YPC^+^ broth was used to study its impact as it contains added nutrients for rapid growth of *H. volcanii* (Strillinger et al. 2016). After biotransformation in the presence of 5 mM acetophenone substrate at 45 °C for 24 h using 20 g of calcium alginate beads with immobilised *H. volcanii*, 57% yield was detected with YPC broth (Fig. 5a). Supplementation of YPC + 4% fructose resulted in a reduction in yield to 47%. Compared to YPC, supplementation with 4% lactate and 4% sucrose resulted in increased yield to 66% and 64% respectively. Maximal 1-phenylethanol production was achieved with 2 × YPC^+^ (95%) and YPC + 4% glucose (98%), which is almost double the yield found with YPC broth only condition.

To determine the optimum temperature for biotransformation, reaction was performed at 25 °C and 45 °C. Growth at 45 °C yields three times more product compared to the reaction conducted at 25 °C (97.5% vs 27%) (Fig. 5b). Agitation speed of the batch culture was also investigated. A comparative analysis of yield at 50 rpm, 100 rpm, 150 rpm and 200 rpm was performed (Fig. 5c). Lowest yield of 47% was found at 50 rpm. Increased yield of 76.5% was found at 100 rpm but maximal product yield of 97% was found at 150 rpm. Interestingly, a lower yield of 65% was detected with the highest speed agitation at 200 rpm. Two different concentrations of acetophenone substrate were compared (Fig. 5d). A yield of 55% was found with 10 mM substrate, which is nearly half of 98% yield found with the 5 mM substrate. Finally, the effect of inducible versus constitutive *adh2* gene expression was tested. Three times more yield was detected from the new constitutively expressing *adh2* strain H3924 (97%) compared to the published tryptophan-induced *adh2* strain H1332 (22%) (Fig. 5e) (Timpson et al. 2013).

### Repeated batch production of 1-phenylethanol by once immobilised *H. volcanii* for 12 successive biotransformation cycles

**Fig. 6:**
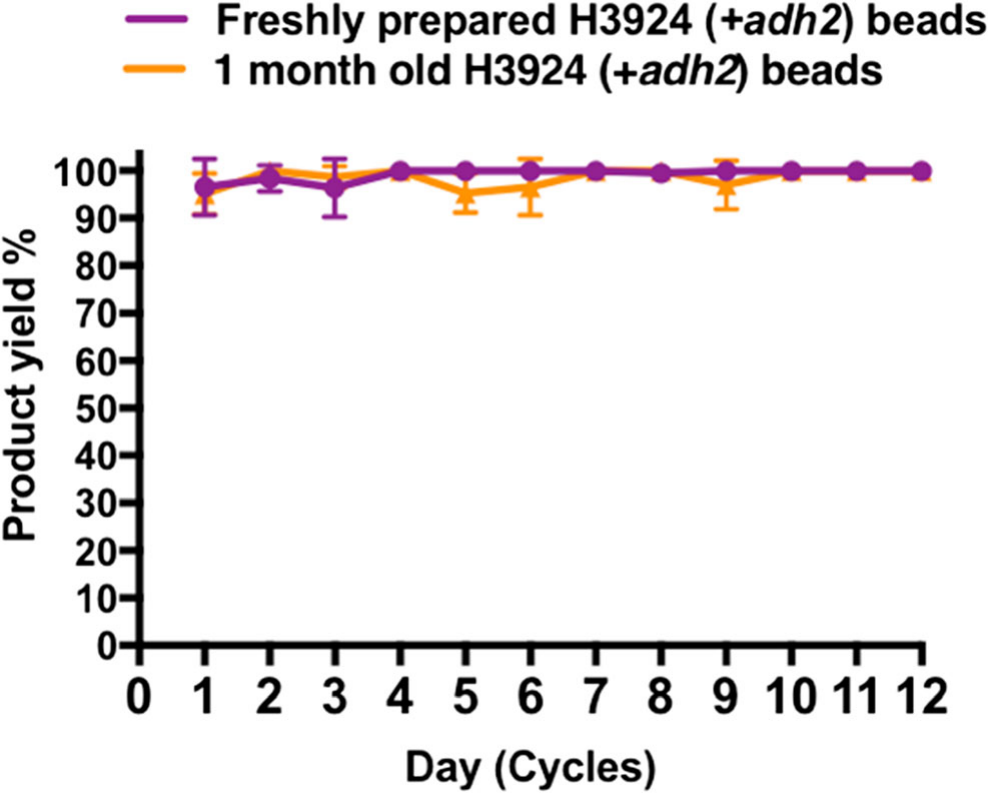
Repeated batch production of 1-phenylethanol by immobilised *H. volcanii* in calcium alginate beads. Product yield was measured by HPLC each day over a successive period of 12 days (cycles). After 24 h, the entire YPC + 4% glucose broth containing beads was decanted, beads were washed twice with 18% salt water and the biotransformation cycle was started again by adding fresh batch of broth supplemented with 5 mM acetophenone substrate. Data represents mean ± SD, *n* = 3

**Fig. 7:**
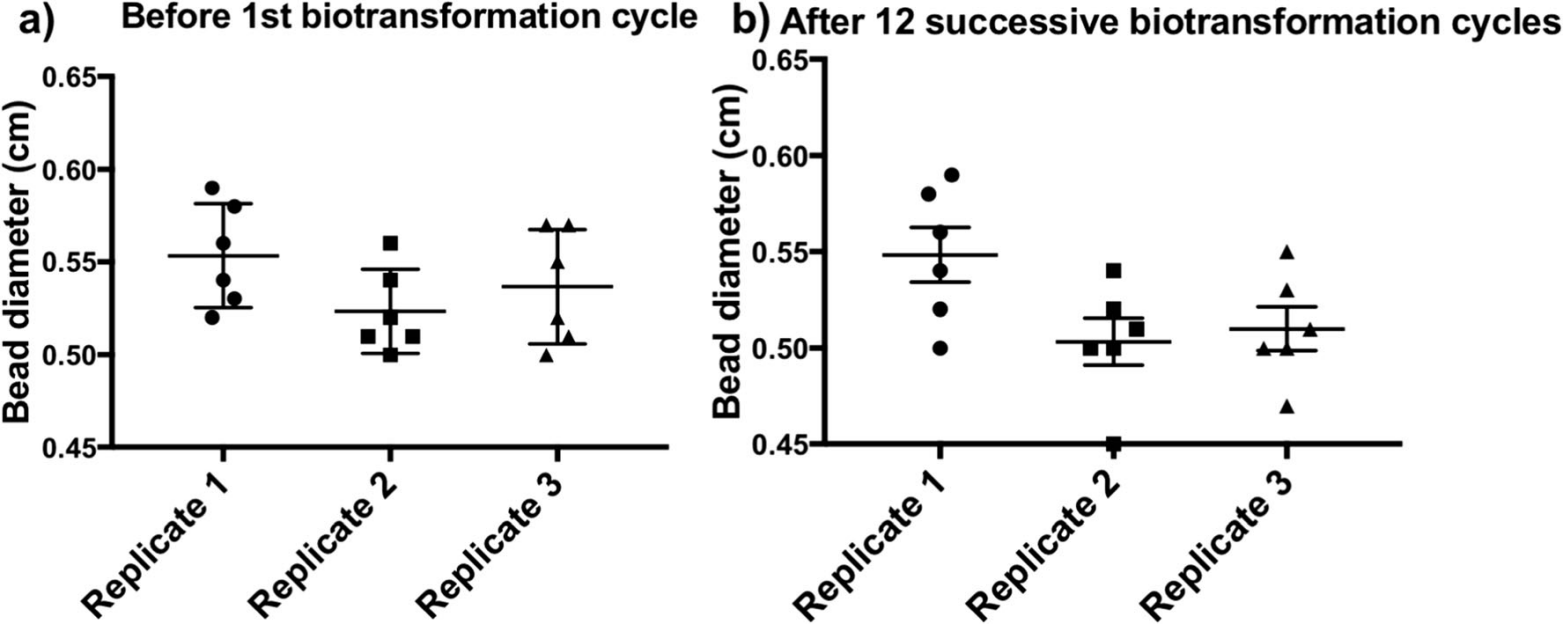
Calcium alginate bead morphology is unperturbed after 12 successive biotransformation cycles. Six beads with immobilised *H. volcanii* were randomly selected from each biological replicate before (**a**) and after 12 biotransformation cycles (**b**). Diameter of the beads were measured, mean ± SD, *n* = 6

The reusability of the system was investigated. Each biotransformation cycle was performed in YPC + 4% glucose broth using once immobilised H3924 whole cells in beads at 45 °C for 24 h and the broth was sampled for 1-phenylethanol detection by HPLC. This process was continued for 12 successive cycles. High 1-phenylethanol production (yield range 96.4–100%) was detected over 12 successive cycles (Fig. 6). No background conversion was detected from the immobilised control strain H3925, which contains the empty vector plasmid pTA1992, and from calcium alginate beads without any immobilised *H. volcanii.* Long-term stability was tested by performing biotransformation using calcium alginate beads with immobilised H3924 that had been stored in 18% salt water solution for a month at room temperature. Repeated batch production of 1-phenylethanol (yield range 95.2–100%) was detected from these beads over successive 12 cycles (Fig. 6). Alginate bead deformation due to bead softening could lead to loss of cells and thereby affect the long-term stability of the whole cell biocatalyst (Smidsrod and Skjak-Braek 1990). Bead diameter was measured before starting the first biotransformation cycle and after the completion of 12 cycles. Data showed that bead diameters were unaffected after 12 successive cycles (Fig. 7a and b).

## Discussion

This work is the first demonstration of *H. volcanii* whole cell–mediated biocatalysis which we exemplified with the reduction of acetophenone to 1-phenylethanol. This system offers halophilic enzyme expression in its native environment, high product yield, reusability and stability. In addition to 1-phenylethanol, the system can be adapted to produce other biotechnologically important compounds from *H. volcanii*. This study is a demonstration of how genetic engineering can be combined with whole cell biocatalysis to design customised system for biotechnology.

Immobilisation within calcium alginate beads makes the system operationally stable and reusable for successive biotransformation cycles. Several steps were essential for producing uniform and stable calcium alginate beads in a reproducible manner. Firstly, when preparing the 4% sodium alginate solution in dH_2_O, sodium alginate powder had to be added very slowly into dH_2_O in small quantity using a magnetic stirrer to prevent clump formation. Any clump will affect the extent of cross-linking and the overall alginate bead size, resulting in poor product yield and operational stability. Secondly, use of a magnetic stirrer to mix *H. volcanii* cells with 4% sodium alginate helps to avoid shear stress on the encapsulated cells and also helps prevent bubble formation in beads. When air bubbles are formed, the alginate bead suspension was left to stand at room temperature for an extra hour to let the bubbles dissolve. Finally, the *H. volcanii*–alginate mixture must be added very slowly in a drop-wise manner into sufficient amount of 1.5% CaCl_2_ (in H_2_O) from a fixed distance (13 cm for this study) to ensure uniform morphology and size of the beads. Any bead partially submerged in CaCl_2_ would have an irregular shape and poor rigidity, which could lead to the leakage of immobilised *H. volcanii*.

It was imperative to strike a balance between rigidity of alginate matrix and porosity, as substrate and product must be transported across the pores to and from the immobilised cells. In principle, any multivalent cation can act as a gelling ion with negatively charged guluronic acid to form alginate beads. The rigidity of the alginate beads depends on the cation used: Ba^2+^ > Sr^2+^ > Ca^2+^ > Pb^2+^ > Cu^2+^ > Ni^2+^ > Cd^2+^ > Zn^2+^ > Co^2+^ > Mn^2+^ (Smidsrød 1974; Strand et al. 2004). Since metal ion–mediated toxicity could be a limiting factor for whole cell–mediated biocatalysis, use of Pb^2+^, Cu^2+^, Cd^2+^, Ni^2+^, Zn^2+^, Co^2+^ and Mn^2+^ was ruled out. Use of high concentrations of Ba^2+^ for gelation could have a negative effect on cells due to the leakage of toxic ions (Mørch et al. 2012). Furthermore beads formed with Ba^2+^and Sr^2+^ are stronger and less porous than beads formed with Ca^2+^ (Morch et al. 2006; Wideroe and Danielsen 2001), and it would be difficult for substrate to access the immobilised cells in a tightly packed and less porous matrix. Ca^2+^ immobilisation was used since it is non-toxic and has been widely used for immobilised whole cell–mediated biocatalysis. Other desirable attributes for an immobilising agent, such as non-reactivity to substrates and products, resistance to microbial degradation, easy preparation and handling and low cost, were also satisfied by this selection.

In YPC broth only condition, a modest yield of 57% was found. Apart from YPC + 4% glucose, similar low yields were found with other supplements such as sucrose, fructose and lactate. The high product yield from YPC + 4% glucose could have been due to glucose bolstering the redox potential of the cells to regenerate the NADPH/NADH cofactor. This is achieved via the dissimilatory metabolism of glucose acting as substrate for NADPH/NADH production (Devaux-Basseguy et al. 1997; Hummel and Gröger 2014). Although ethanol could have been used as an alternative cosubstrate for cofactor regeneration (Alsafadi et al. 2017; Kometani et al. 1995), it could have reacted with acetophenone substrate and would have been toxic for the *H. volcanii* whole cell biocatalyst. Furthermore, glucose can serve as cosubstrate for either NADH- or NADPH-dependent bioreduction under both aerobic and anaerobic conditions, whereas ethanol cannot serve as cosubstrate for NADPH-dependent bioreduction in anaerobic conditions (Kometani et al. 1994). *Hv*ADH2 activity is predominantly NADPH-dependent (Timpson et al. 2013). High yield was also seen with 2 × YPC^+^ broth and could have been due to the increased growth of *H. volcanii*, since this broth contains additional yeast extract, casamino acid, lactate, K_2_HPO_4_/KH_2_PO_4_ and NH_4_Cl nutrients (Strillinger et al. 2016). YPC + 4% glucose broth was chosen as it is more economical than the costs associated with supplementation of the 2 × YPC^+^ broth.

Higher yield at 45 °C compared to 25 °C was due to *H. volcanii* growing optimally at 45 °C. This is in keeping with previous whole cell biocatalysis studies where organism’s temperature optimum yields the highest 1-phenylethanol product (Homola et al. 2015; Illeová et al. 2015; Kurbanoglu et al. 2010). Lowest yield found at 50 rpm was probably due to slow agitation resulting in insufficient contact between substrate and biocatalyst. Maximal product yield using *H. volcanii* requires effective contact between substrate and biocatalyst, as well as effective aeration of the immobilised cells. To this end, agitation at 150 rpm appeared to provide the right balance required for maximal product yield. We also noticed some debris in the media resulting from bead disintegration with repeated agitation at 200 rpm. Multiple explanations could account for a significant drop in product yield to ~ 50% with 10 mM substrate (compared to 5 mM substrate). Firstly, excessive substrate (10 mM) might lead to sufficient accumulation of product (~ 5 mM) that interferes with the reaction, leading to reduced *Hv*ADH2 activity. A study using yeast *Rhodotorula glutinis* whole cells reported inhibition of biocatalyst by product accumulation (Valadez-Blanco and Livingston 2009). This problem could be overcome by *in situ* product removal from the biocatalyst (Freeman et al. 1993). Secondly, excessive substrate (10 mM) might be toxic and compromise the performance of the whole cell biocatalyst. Intermittent substrate feeding strategy could be used to overcome this issue (Valadez-Blanco and Livingston 2009).

Existing gene expression systems for *H. volcanii* are based on a tryptophan inducible promoter (p.*tna*) (Allers 2010). The constitutive expression system developed in this study alleviates the need for any tryptophan supplementation (which may result in batch-to-batch variation) and also improves the product yield significantly compared to tryptophan-inducible expression system. Furthermore, repeated supplementation of tryptophan would not be cost effective since it is an expensive amino acid. However, use of this strong constitutive promoter has to be carefully considered when overexpression of the enzyme is toxic for cells.

Our whole cell immobilisation method generated roughly 60 g of beads from 3.5 g of *H. volcanii* cell pellet (380 ml broth culture), of which 20 g were used in all biotransformation reactions. Therefore, in this system, 1.1 g dry weight (120 ml broth culture) of whole cell biocatalyst had a high product yield range of 96.4–100% under optimal conditions (in the presence of 5 mM acetophenone substrate after 24 h). By comparison, 5 g of *Aspergillus niger* whole cell biocatalyst was reported to show a yield range of 53–63% in the presence of 1 mM acetophenone substrate, after 24 h (Kurbanoglu et al. 2007). In another study, 500 ml of *Yarrowia lipolytica* gave a yield range of 48–94% in the presence of 3.3 mM substrate, after 48 h (Janeczko et al. 2015). The performance of our whole cell biocatalyst is several-fold greater than the above two studies. Furthermore, we provide evidence for high product yield using whole cell biocatalyst for 12 successive cycles, both from freshly prepared beads as well as beads stored at room temperature for a month. In this system, any 1-phenylethanol detected is solely due to the *Hv*ADH2 activity from the immobilised H3924 cells, since there was no background conversion in the control strain H3925, or in alginate beads without any immobilised *H. volcanii*. The engineered strain H1325 (*Δadh2 Δadh1 ΔpyrE2 ΔhdrB Nph-pitA Δmrr*) used in this work is depleted of all endogenous ADH activity and bioconversion is detected only when the recombinant *Hv*ADH2 is expressed.

Unchanged diameters of calcium alginate beads confirmed their stability. It is noteworthy the YPC broth contains 2.46 M Na^+^, 0.18 M Mg^2+^ and 3 mM Ca^2+^ of which Na^+^ and Mg^2+^ have been referred to as anti-gelling cations for alginate beads (Melvik and Dornish 2004). However, several studies indicate that Mg^2+^ acts as a cross-linker for alginate bead formation (Topuz et al. 2012; Vicini et al. 2017). Multiple explanations could account for alginate beads in this system withstanding molar concentrations of anti-gelling cations. Firstly, high alginate concentration (4%) meant there was abundance of guluronic acid residues which make gels stable in the presence of monovalent cations such as Na^+^ (Martinsen et al. 1989). High guluronic acid content also transforms Mg^2+^ into a gelling cation (Topuz et al. 2012). Secondly, the presence of 3 mM Ca^2+^ in the YPC broth would have contributed to the stability of the calcium alginate beads, as supplementation of mM calcium is essential for the maintenance of alginate beads (Strand et al. 2004). A 3 mM Ca^2+^ is also present in 18% salt water used in bead washing steps, this is necessary as *H. volcanii* cells lyse in a low salt environment.

This system can easily be scaled up by packing alginate beads with immobilised *H. volcanii* cells into a controlled stirred tank bioreactor (Strillinger et al. 2016). Instead of batch culture condition, it can also be used in a continuous flow system for increased productivity (Porta et al. 2016; Tamborini et al. 2018; Tamborini et al. 2013). When compared with other extremophiles such as thermophiles, the biotechnological potential of halophiles has been less explored. Production of β-carotene by green algae *Dunaliella salina* and ectoine, an enzyme immobiliser used in cosmetic products by *Halomonas elongata* represent the few examples of successful application of halophiles in industrial biotechnology (Oren 2010). This could be due to halophiles such as *H. volcanii* being mechanically fragile and prone to lysis with decreased salt concentrations. This development of this system of immobilised cells in alginate beads circumvents some of these issues and harnesses the biotechnological potential of halophiles to produce valuable biomolecules.
